# Exploring the extremes: applying high concentration of yeast extract leads to drastic morphological changes and elimination of (+)-geodin and asterric acid production in *Aspergillus terreus* submerged cultures

**DOI:** 10.1007/s10529-020-03018-5

**Published:** 2020-10-07

**Authors:** Tomasz Boruta, Adrianna Górnicka, Iwona Grzybowska, Ida Stefaniak, Marcin Bizukojć

**Affiliations:** Faculty of Process and Environmental Engineering, Department of Bioprocess Engineering, Lodz University of Technology, ul. Wolczanska 213, 90-924 Lodz, Poland

**Keywords:** *Aspergillus terreus*, Fungal morphology, Lovastatin, Secondary metabolites, Yeast extract

## Abstract

**Objective:**

Evaluation of morphology and secondary metabolites production in *Aspergillus terreus* ATCC 20542 cultures over a wide range of lactose and yeast extract concentrations from 0.2 up to an extremely high level of 200 g l^−l^.

**Results:**

The morphological differences of mycelial objects were quantified with the use of morphological parameters calculated by applying the tools of digital image analysis. At 200 g l^−l^ of yeast extract clumps and loose hyphae were recorded instead of pellets commonly observed in submerged cultures of *A. terreus*. Under these conditions the biosynthesis of (+)-geodin and asterric acid was totally blocked, lovastatin formation was found to be at a relatively low level and biomass production turned out to be greater than in the remaining variants, where the pelleted growth was observed. At 200 g l^−l^ of lactose the production of lovastatin, (+)-geodin and asterric acid was visibly stimulated compared to the media containing 0.2, 2 and 20 g l^−l^ of the sugar substrate, but at the same time no traces of butyrolactone I could be detected in the broth. Lactose at the extremely high concentration of 200 g l^−l^ did not induce the drastic morphological changes observed in the case of 200 g l^-1^ of yeast extract. It was proved that at the C/N values as low as 4 and as high as 374 *A. terreus* not only continued to display growth but also exhibited the production of secondary metabolites. The use of cultivation media representing the equivalent C/N ratios led to different metabolic and morphological outcomes depending on the concentration of lactose and yeast extract that contributed to the given C/N value.

**Conclusion:**

The extremely high concentration of yeast extract leads to marked morphological changes of *A. terreus* and the elimination of (+)-geodin and asterric production, while applying the excess of lactose is stimulatory in terms of lovastatin production.

**Electronic supplementry material:**

The online version of this article (10.1007/s10529-020-03018-5) contains supplementary material, which is available to authorized users.

## Introduction

The repertoire of fungal secondary metabolites is a rich source of bioactive substances of potential pharmaceutical interest (Adrio and Demain 2003). In contrast to primary metabolites, which are essential for the growth and energy processes of the cell, the roles of secondary metabolites are rather associated with specific ecological functions and often remain unclear (Brakhage 2013; Keller 2015). Importantly, the induction of secondary metabolic pathways proceeds in response to countless environmental signals. It has been shown previously that performing the cultivation in non-standard media may be an effective approach to influence the fungal biosynthetic routes, e.g. by using extremely high salt concentrations to trigger the stress-related cellular mechanisms (Wang et al. 2011; Jančič et al. 2016; Overy et al. 2017).

Lovastatin, a cholesterol-lowering drug representing the biosynthetic family of polyketides, serves as a textbook example of a fungal metabolite with a significant medical impact (Alberts et al. 1980). Its industrial-scale production is based on the submerged cultivation of high-yielding mutants of *Aspergillus terreus*. The efforts aimed at maximizing the yield and productivity of lovastatin are basically centered around the optimization of fungal strains, medium composition, cultivation conditions and, importantly, morphological development (Mulder et al. 2015). Many of the lovastatin-overproducing fungi were derived via mutagenesis of the reference wild-type strain *A. terreus* ATCC 20542 (Vinci et al. 1991; Kennedy et al. 1999). It was previously demonstrated that the catalog of secondary metabolites biosynthesized by this particular strain under submerged conditions includes not only lovastatin, but also (+)-geodin, asterric acid and butyrolactone I (Boruta and Bizukojc 2016). Previous studies revealed that (+)-geodin displays antiviral and antimicrobial activities (Rinderknecht et al. 1947; Takatsuki et al. 1969). It also acts as a glucose uptake stimulator towards rat adipocytes and enhances the fibrinolytic activity of vascular endothelial cells (Shinohara et al. 2000; Sato et al. 2005). Asterric acid and butyrolactone I exhibit the activities of endothelin binding inhibitor (Ohashi et al. 1992) and cyclin-dependent kinases inhibitor, respectively (Kitagawa et al. 1994). Until now, the influence of medium composition on the production of multiple secondary metabolites in relation to the morphological parameters has not been studied for *A. terreus* cultures. Moreover, the response of the metabolism and morphology of *A. terreus* to extremely high levels of key medium components, namely the carbon and nitrogen sources, have not been investigated so far.

The aim of the present study was to evaluate the production of secondary metabolites and quantitatively characterize the morphological development in the submerged cultures of *A. terreus* ATCC 20542 over a wide range of concentrations of carbon source (lactose) and nitrogen source (yeast extract), i.e. from 0.2 g l^−l^ up to an extremely high level of 200 g l^−l^.

## Materials and methods

### Strain

*Aspergillus terreus* ATCC 20542, a strain purchased from the *American Type Culture Collection* (ATCC), was used throughout the study and maintained on agar slants according to the instructions provided by ATCC.

### Cultivation media

**Fig. 1:**
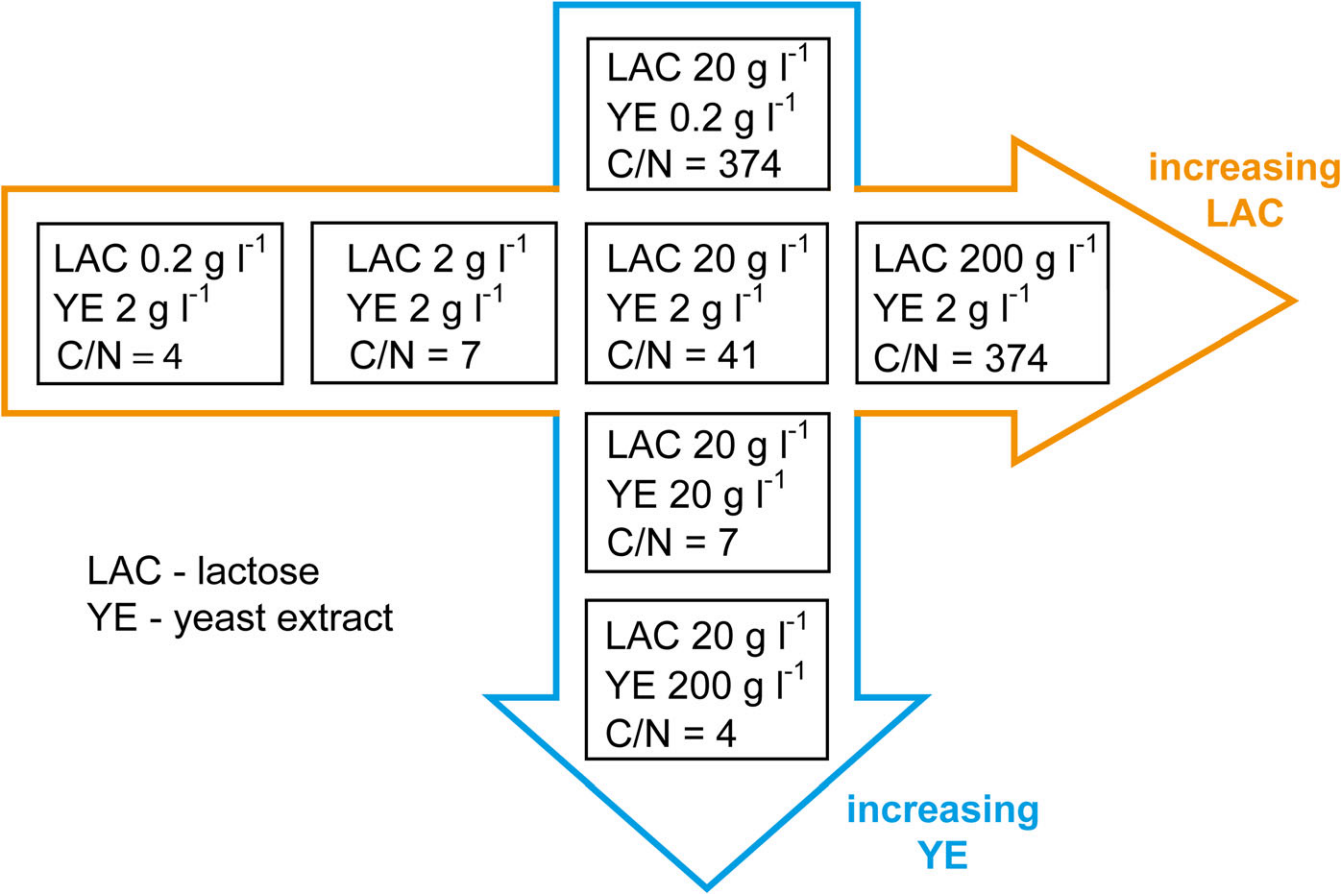
Schematic illustration of seven cultivation media used in the present study. The concentration values of lactose (LAC), yeast extract (YE) and the C/N ratio are indicated for each medium. The media are ordered with respect to the increasing LAC and YE concentrations

The experiment involved seven variants of the cultivation medium that differed with respect to the concentration of lactose and yeast extract and thus represented a wide range of C/N ratios, as shown schematically in Fig. 1. Apart from lactose (weighed as lactose monohydrate) and yeast extract the media contained the following ingredients: KH_2_PO_4_ 1.51 g l^−1^, MgSO_4_·7H_2_O 0.51 g l^−1^, NaCl 0.4 g l^−1^, biotin 0.04 mg l^−1^ and 1 ml per 1 liter of the medium of the following solution: CuSO_4_·5H_2_O 250 mg l^−1^, ZnSO4·7H_2_O 1 g l^−1^, Fe(NO_3_)_3_·9H_2_O 2 g l^−1^, MnSO_4_ 50 mg l^−1^, Na_2_B_4_O_7_·10H_2_O 100 mg l^−1^, Na_2_MoO_4_·2H_2_O 50 mg l^−1^. Yeast extract was purchased from Becton Dickinson (USA). The initial C/N ratio for each medium (presented in Fig. 1) was estimated according to C- and N-contents of lactose and yeast extract given by Casas Lopez et al. (2003). All media were autoclaved at 121 °C after adjusting the pH to 6.5 with the use of NaOH solution.

### Growth conditions

Cultivation was performed in flat-bottomed shake flasks with a total volume equal to 500 ml and a working volume of 150 ml. The flasks were inoculated with 10^9^ spores per liter of the medium. The spores were washed from agar slants with the use of a sterile plastic pipette at the moment of inoculation. The cultures were propagated as 1-stage runs (without preculture). The flasks were incubated for 168 h in Certomat^®^ BS-1 (B. Braun Biotech International, Germany) rotary shaker at 110 min^-1^. The temperature was kept constant at 28 °C throughout the experiment. All cultivation runs were performed in triplicates. Aseptic conditions were maintained to prevent microbial contamination.

### Analytical procedures

The 40 ml samples were filtered through a paper filter and the filtrates were stored at – 20 °C. Biomass production was determined by weighting the filter with biomass after drying at 105 °C to constant mass. The concentration of lactose and lovastatin was determined as described in the previous work (Bizukojc et al. 2012). Total nitrogen was assayed with the use of IL550TOC-TN analyzer (HACH, USA) according to the manufacturer’s instructions. The identification and quantification of secondary metabolites in the liquid samples was performed with the use of ultra-performance liquid chromatography coupled with high-resolution mass spectrometry (UPLC-MS) system (Waters, USA) as previously described (Boruta and Bizukojc 2016). The analytical standard solution of lovastatin in its β-hydroxy acid form was obtained from lovastatin lactone according to the procedure described by Casas Lopez et al. (2003). Microscopic analysis was performed with the use of Olympus BX53 microscope. Digital image analysis was carried out according to Kowalska et al. (2018) with the use of Olympus cellSens Dimension Desktop 1.16 (Olympus, Japan). Four morphological parameters were determined for at least 40 individual objects at any considered time point, namely the projected area (pixel count of an object multiplied by a squared unit of calibration), elongation (the squared quotient of longitudinal and transversal deviation of all pixels that belong to a given object along the regression line), convexity (ratio of projected area to convex area) and circularity (the area relative to the area of a circle with an equal perimeter) (Kowalska et al. 2018).

### Statistical analysis

The cultivation process was performed in triplicates. For each tested variant, the medium was prepared in three individual batches and distributed to three sets of flat-bottom flasks prior to sterilization. Each flask was inoculated separately after autoclaving. During the experiment, three samples were drawn for each tested medium variant in 24-h intervals (one sample per flask). The samples were then analyzed to determine the concentration of metabolites, lactose and glucose and the production of biomass. The standard deviation was calculated for the triplicates with the use of OriginPro software (OriginLab, USA) and the results were reported as “mean concentration ± standard deviation”. In the case of the morphological parameters calculated on the basis of microscopic observations, namely the projected area, convexity and circularity, the mean and standard deviation values corresponded to the measurements made for at least 40 mycelial objects (Kowalska et al. 2018). The two-sample t-test (significance level α = 0.05) was performed with the use of OriginPro software to evaluate, whether the mean results obtained for the standard cultivation medium (containing 20 g l^−1^ of lactose and 2 g l^−1^ of yeast extract) differed significantly from the mean values recorded for the remaining tested media. The *P* values resulting from the two-sample *t* test are presented in Supplementary Tables 1–11.

## Results and discussion

**Fig. 2:**
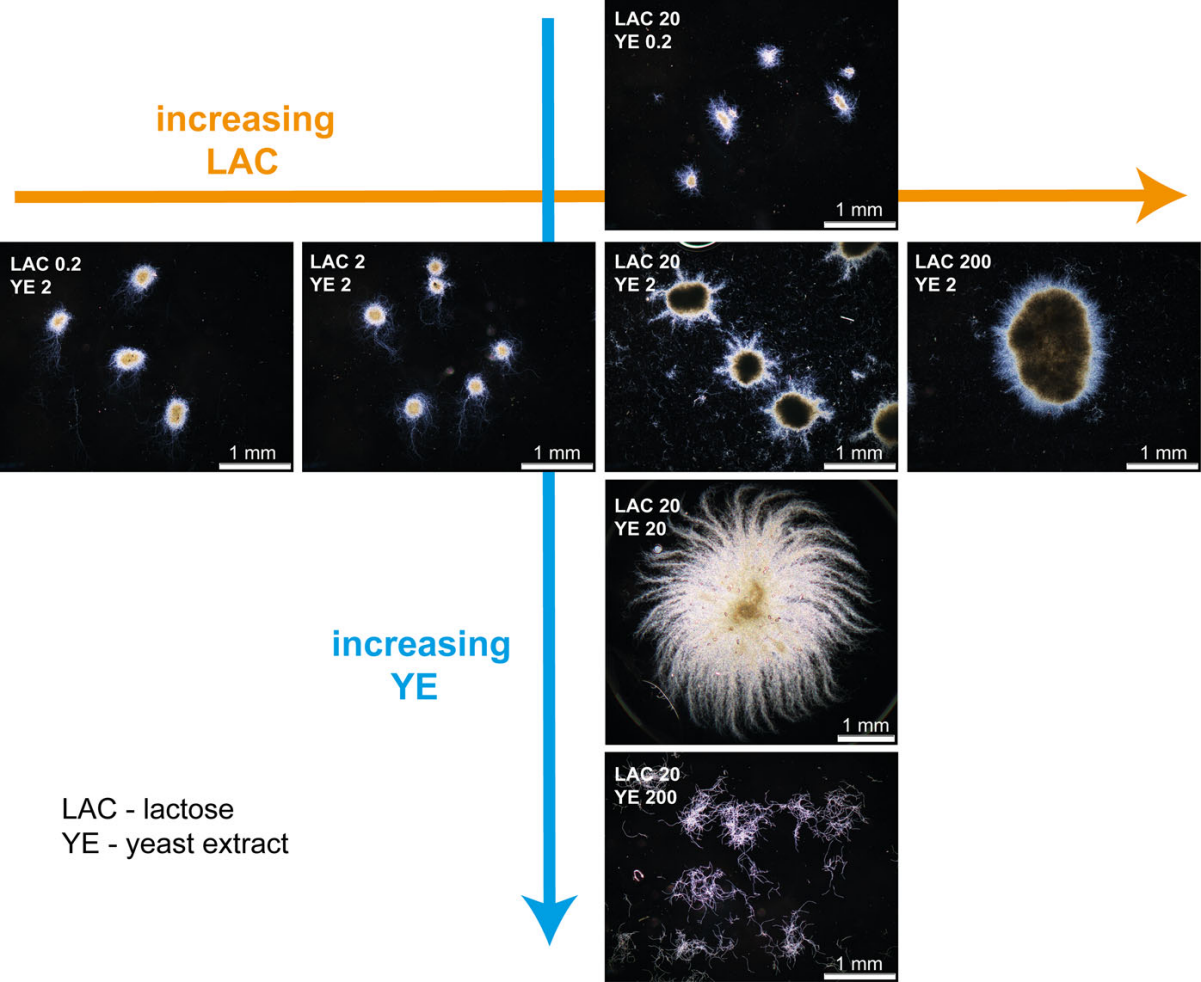
Microscopic images of the morphological forms of *A. terreus* ATCC 20542 observed in the tested cultivation media. The concentration values of lactose (LAC) and yeast extract (YE) are indicated in g l^−1^ for each medium. The images are ordered with respect to the increasing LAC and YE concentrations. The presented microscopic images were taken at 168 h of the cultivation process. Scale bar represents 1 mm

**Fig. 3:**
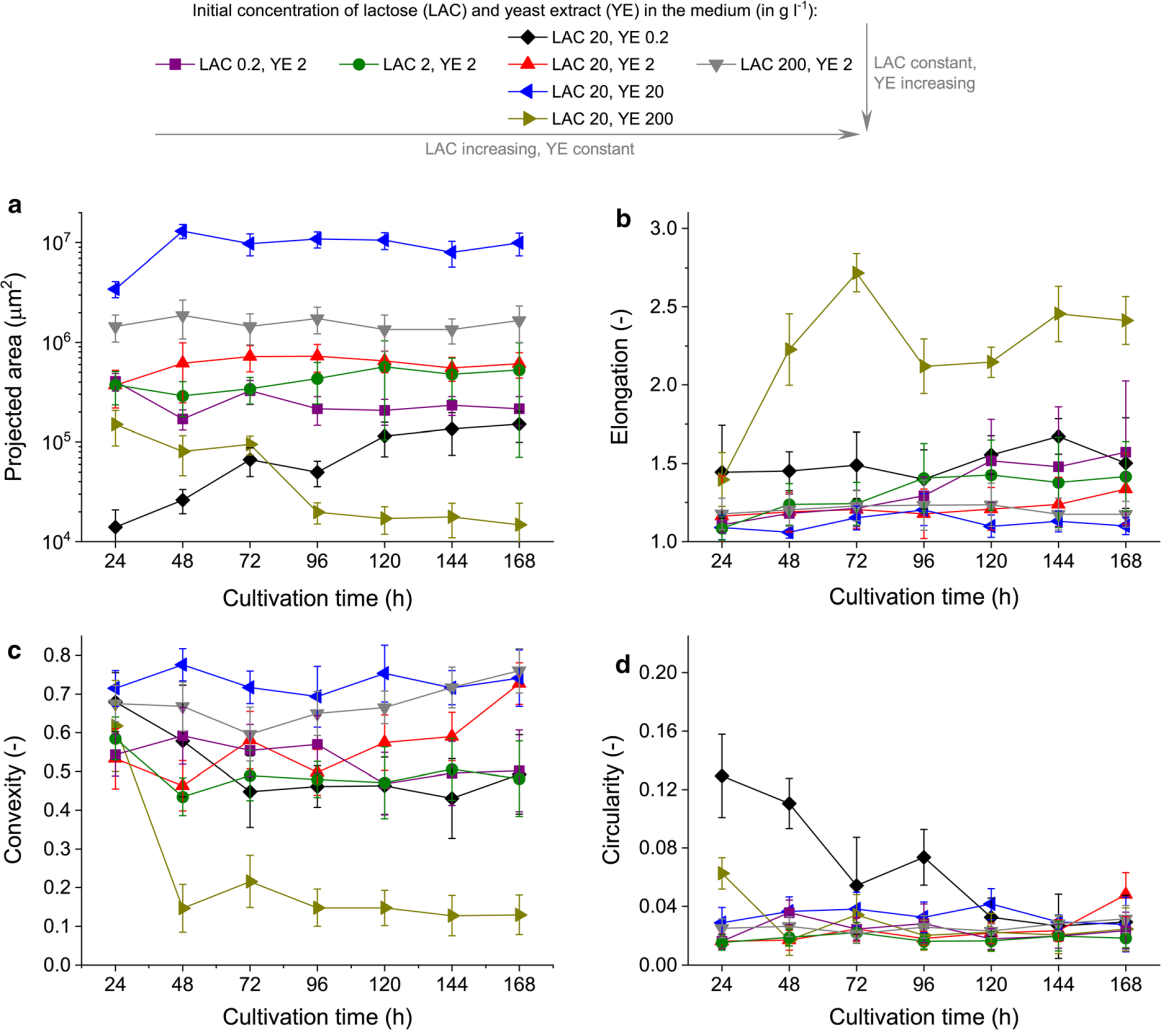
Time courses of **a** projected area, **b** elongation, **c** convexity and **d** circularity values determined for *A. terreus* ATCC 20542 in the tested cultivation media. Each data point was obtained by analyzing 40 objects sampled from cultivation triplicates and the corresponding standard deviation values are given as error bars. The *P* values resulting from the two-sample t-test are presented in Supplementary Tables 1–4 to indicate, whether the mean results obtained for the standard cultivation medium (containing 20 g l^−1^ of lactose and 2 g l^−1^ of yeast extract) differed significantly from the mean values recorded for the remaining tested media

**Fig. 4:**
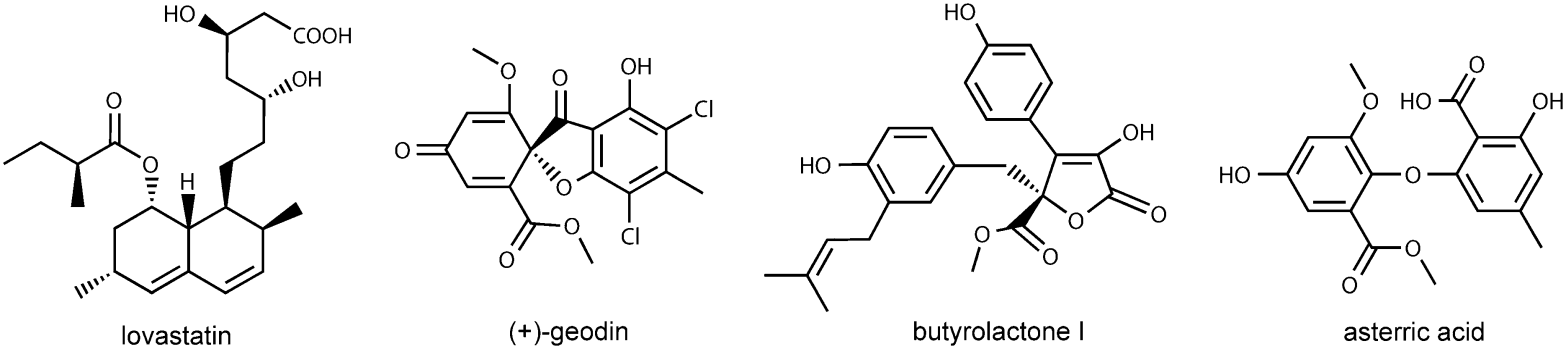
Structural formulae of secondary metabolites of *A. terreus* ATCC 20542 detected and quantified in the present study

**Fig. 5:**
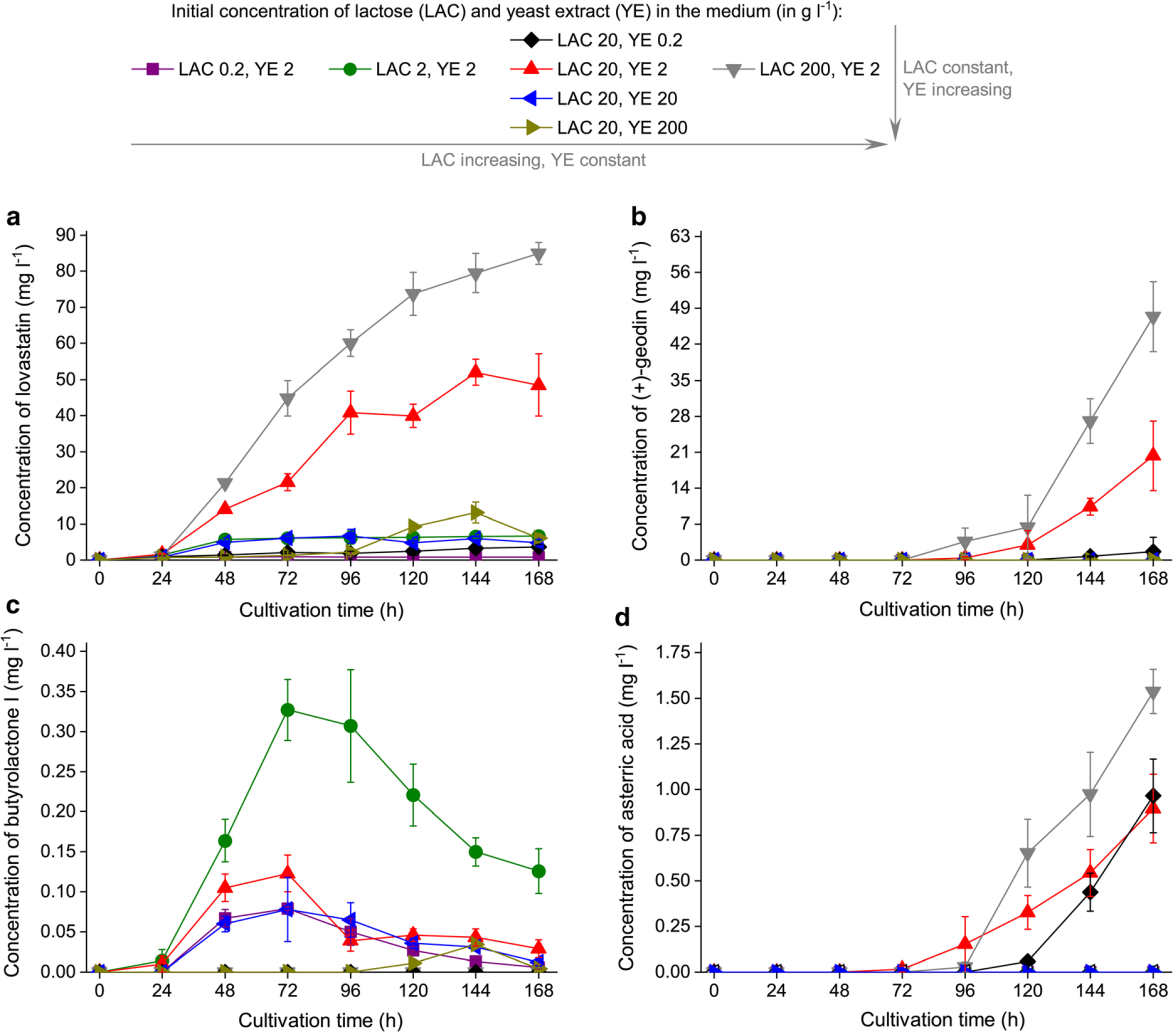
Time courses of **a** lovastatin, **b** (+)-geodin, **c** butyrolactone I and **d** asterric acid concentration values in *A. terreus* ATCC 20542 cultures propagated in the tested cultivation media. Cultivation runs were performed in triplicates and the corresponding standard deviation values are reported as error bars. The *P* values resulting from the two-sample *t* test are presented in Supplementary Tables 5–8 to indicate, whether the mean results obtained for the standard cultivation medium (containing 20 g l^−1^ of lactose and 2 g l^−1^ of yeast extract) differed significantly from the mean values recorded for the remaining tested media

**Fig. 6:**
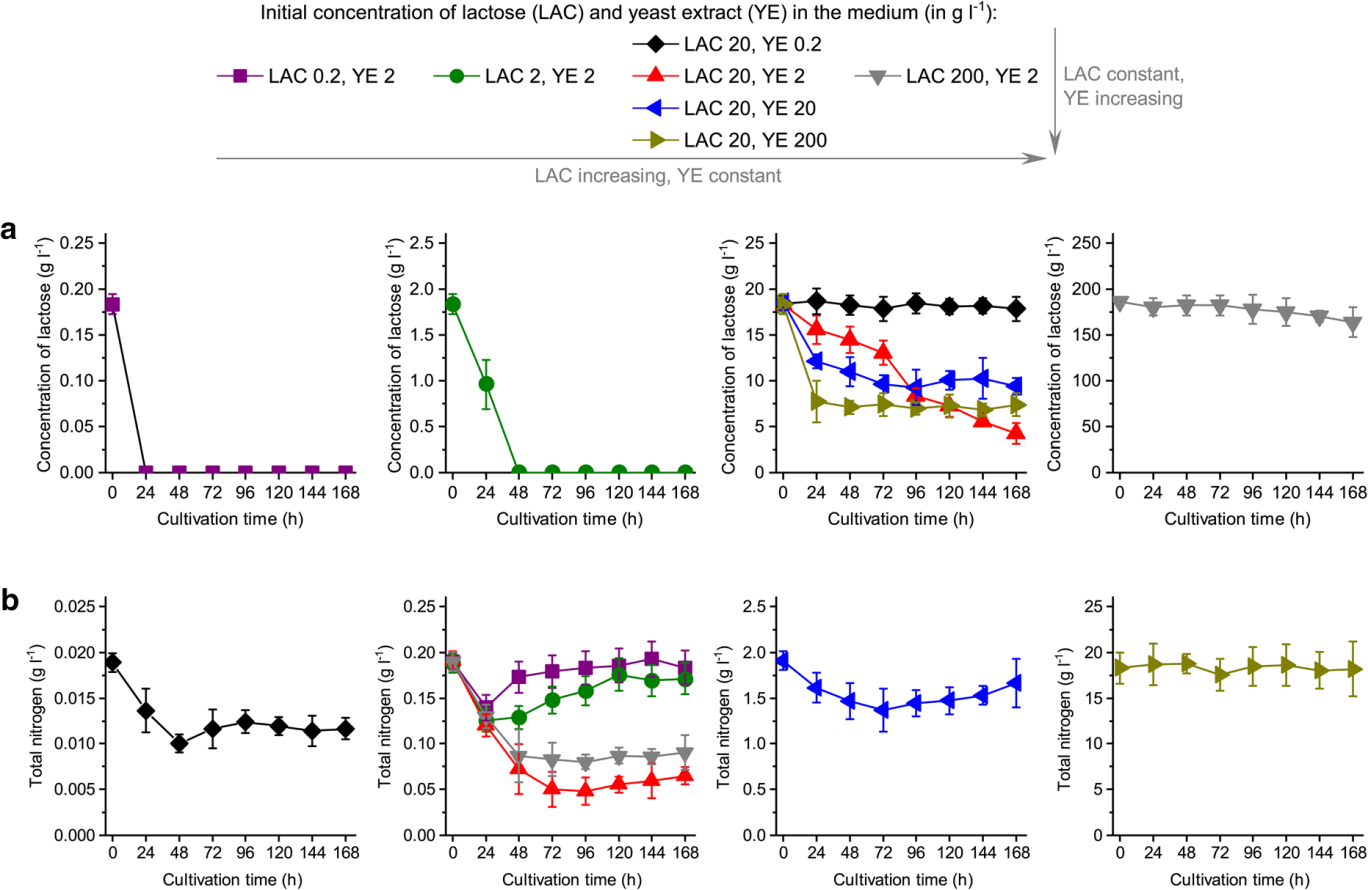
Time courses of (**a**) lactose and (**b**) total nitrogen levels in *A. terreus* ATCC 20542 cultures propagated in the tested cultivation media. Cultivation runs were performed in triplicates and the corresponding standard deviation values are reported as error bars. The *P* values resulting from the two-sample *t* test are presented in Supplementary Tables 9 and 10 to indicate, whether the mean results obtained for the standard cultivation medium (containing 20 g l^−1^ of lactose and 2 g l^−1^ of yeast extract) differed significantly from the mean values recorded for the remaining tested media

**Fig. 7:**
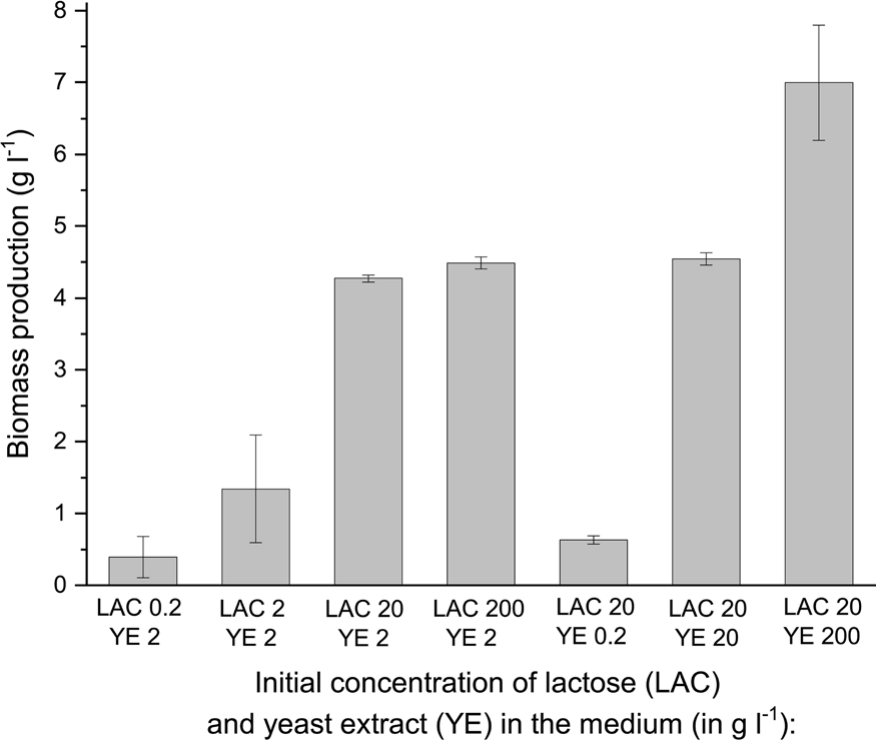
Biomass production in *A. terreus* ATCC 20542 cultures propagated in the tested cultivation media. The samples were collected at 168 h of cultivation. Cultivation runs were performed in triplicates and the corresponding standard deviation values are reported as error bars. The *P* values resulting from the two-sample t-test are presented in Supplementary Table 11 to indicate, whether the mean results obtained for the standard cultivation medium (containing 20 g l^−1^ of lactose and 2 g l^−1^ of yeast extract) differed significantly from the mean values recorded for the remaining tested media

The cultivation of *A. terreus* ATCC 20542 was performed in seven distinct growth media differing with respect to lactose (hereinafter referred to as LAC) and yeast extract (YE) concentrations and thus representing various C/N ratios. The initial levels of YE and LAC ranged from 0.2 to 200 g l^-1^, as shown schematically in Fig. 1. Among the tested variants, the standard formulation containing 20 g l^−1^ of LAC and 2 g l^−1^ of YE was taken directly from literature (Boruta and Bizukojc 2016), whereas the remaining ones were suggested in the current work. Here, the concentration of 200 g l^−1^ was considered to be an “extremely high” substrate level, chosen by considering the concentration limits of carbon and nitrogen sources in the previously described *A. terreus* cultivations (reviewed by Mulder et al. 2015). The outcomes of the cultivation runs were evaluated with respect to fungal morphology (Figs. 2 and 3), secondary metabolites production (Figs. 4 and 5), levels of lactose and total nitrogen in the medium (Fig. 6) and biomass production (Fig. 7).

The visual inspection of shake flasks and daily microscopic observations of mycelia revealed that altering the initial concentration of YE in the medium had a profound effect on the development of morphological forms of *A. terreus* (Fig. 2). Increasing the YE levels from 0.2 up to 20 g l^−1^ resulted in the marked differences with respect to the size of the pellets. The higher the concentration of YE, the larger the pellets were observed in the broth. At 20 g l^−1^ the remarkably fluffy, loose and large pellets resembling the “exploding fireworks” were recorded, with a diameter exceeding 3 mm (Fig. 2). Interestingly, the “more YE leads to larger pellets” trend was not maintained when the YE level was increased from 20 to 200 g l^−1^, as it surprisingly turned out that at 200 g l^−1^ of YE *A. terreus* proliferated not as mycelial pellets, but in the form of clumps and loose hyphae (Fig. 2). Such a drastic medium-dependent transition from pelleted to dispersed morphology in *A. terreus* cultures has never been observed before. From a bioprocess engineering perspective, the morphological transition between dispersed and pelleted morphology has profound effects on the rheological properties of the broth, oxygen transfer, mixing, substrates diffusion and energy requirements of the cultivation run (Posch et al. 2013). The reasons of the morphological transition observed in the present study were not clear, but it cannot be excluded that the extremely high content of charged molecules present in YE, mostly amino acids, affected the surface charges of fungal spores and thus disturbed the pellet formation mechanism, which is very much dependent on the attraction and repulsion forces associated with the charge of the spores (Veiter et al. 2018). The consequences of increasing LAC levels were not as striking as in the case of YE (Fig. 2). It was noted that LAC, even at extremely high concentrations, did not influence the morphological development of *A. terreus* as radically as YE, possibly due to the fact that lactose molecules are not charged and do not disable the typically observed spore agglomeration events responsible for the development of fungal pellets. The pelleted morphology was maintained regardless of LAC concentration in the medium, albeit the differences in pellets size were clearly visible (Fig. 2). It is also worth mentioning that at the lowest applied concentrations of LAC and YE equal to 0.2 g l^−1^ the growth of the fungus was recorded but the pellets were clearly much smaller than in the nutritionally richer media (Fig. 2).

After the preliminary visual inspection of microscopic images, the morphological outcomes of increasing YE and LAC concentrations were evaluated quantitatively within the computational framework of digital image analysis. The evolution of mycelial objects was monitored and compared with the use of the following morphological parameters: projected area (Fig. 3a), elongation (Fig. 3b), convexity (Fig. 3c) and circularity (Fig. 3d). The values of the projected area (Fig. 3a) increased in a step-by-step fashion, each time by an order of magnitude when the initial level of YE rose from 0.2 to 2 and then to 20 g l^−1^. This was a direct reflection of the fact that the size of pellets clearly increased within this interval of YE concentrations (see Fig. 2). Then, a sharp decrease in projected area values took place when the YE level changed from 20 to 200 g l^−1^ (Fig. 3a). As mentioned before, this was associated with the morphological transition from pelleted to clumped and loose morphology (Fig. 2). Furthermore, the analysis revealed that the average area of clumps in the “200 g l^−1^” variant decreased visibly after 72 h of the run (Fig. 3a). The highest-YE variant stood out not only in terms of the projected area but also with respect to elongation (Fig. 3b) and convexity (Fig. 3c). The values of these two morphological parameters recorded for the medium containing 200 g l^−1^ of YE differed substantially compared to other tested variants. The morphological behavior of *A. terreus* ATCC 20542 in this medium can be regarded as exceptional, as this strain is known to proliferate in the form of pellets under the conditions of submerged cultivation (see the review of Bizukojc and Ledakowicz (2015) and references therein). As far as the variations in lactose concentration were concerned, the differences in pellet size observed in the microscopic images were reflected by the values of the projected area (Fig. 3a), however the differences between the LAC variants were definitely not as striking as for the varying YE levels. Also in the case of other monitored parameters (Fig. 3b–d), increasing the concentration of LAC from 0.2 up to 200 g l^−1^ did not lead to as remarkable morphological changes as in the case of the investigated YE counterparts.

During the course of the cultivation four major secondary metabolites of *A. terreus* (Fig. 4) were detected in the broth and quantified, namely lovastatin (in its β-hydroxy acid form) (Fig. 5a), (+)-geodin (Fig. 5b), butyrolactone I (Fig. 5c) and asterric acid (Fig. 5d). The consequences of applying an extremely high concentration of LAC or YE were markedly different with respect to the biosynthetic capabilities of the examined strain. The high-YE variants (20 and 200 g l^−1^ of YE) turned out to be effective in terms of totally eliminating the main by-products typically found during the lovastatin-oriented cultivations, namely (+)-geodin and asterric acid, but the levels of lovastatin were low in this case, reaching only 13 mg l^−1^. On the other hand, under extremely high LAC concentration the biosynthesis of butyrolatone I was completely blocked and the production of the target metabolite was boosted to reach 85 mg l^−1^, but at the cost of relatively high (+)-geodin and asterric acid titers equal to 47 and 1.5 mg l^−1^, respectively. Regarding the variants with lowest levels of LAC and YE, the production of the majority of the considered secondary metabolites was barely visible in these cases. Traces of lovastatin were present, but (+)-geodin and asterric acid could not be found at all. An exception was recorded in relation to butyrolactone I production, where the time course recorded for the “0.2 g l^−1^ of LAC/2 g l^−1^ of YE” medium resembled the one observed for the “20 g l^−1^ of LAC/20 g l^−1^ of YE” variant. Hence, even under the applied conditions of poor substrate availability some of the secondary metabolic pathways were still in an active state. Many of the previously published studies focused entirely on the aspect of lovastatin formation, while not addressing the production of secondary metabolites from a broader perspective to include more products in the analysis. As it is not uncommon for the fungal secondary metabolites to display bioactive properties which are not fully characterized with respect to possible physiological impacts, gathering data on (+)-geodin, asterric acid butyroloactone I production is justified in the context of possible future studies, including the ones related to drug discovery. Furthermore, one should not ignore the impact of medium composition on the biosynthesis of these metabolites, as they are known to be the major by-products of lovastatin-oriented cultivations and need to be eliminated in the series of expensive downstream processing steps to ensure the required purity of the target molecule.

Apart from morphological analysis and time courses of metabolites concentration the levels of LAC (Fig. 6a) and total nitrogen (Fig. 6b) were analyzed. In the media with 0.2 and 2 g l^−1^ of LAC the sugar was totally depleted at 24 and 48 h of the run, respectively. When 20 g l^−1^ of LAC was applied, the substrate was still available even at the very end of the cultivation (after 168 h). Depending on the variant, the distinct time courses of LAC levels were recorded (Fig. 6a). At the initial YE concentration equal to 0.2 g l^−1^ the consumption of lactose was barely observable. The higher the YE level, the faster was the LAC consumption during the first 24 hours of the run. However, during the remaining days of the experiment the opposite correlation was observed, namely at 20 and 200 g l^−1^ of YE the utilization of LAC was practically negligible between 24 and 168 h, whereas at 2 g l^−1^ it continued to proceed until the end of cultivation (Fig. 6a). Total nitrogen analysis (Fig. 6b) revealed that nitrogen was never totally depleted in the medium regardless of the variant. The lowest residual concentration was noted for the medium containing 20 g l^−1^ of LAC. Moreover, it was not uncommon to observe the gradual decrease of nitrogen level until a certain time point, when the time course of total nitrogen levels started to exhibit an increasing trend. Since all the measurements were conducted with respect to the cultivation broth, not the intracellular environment, one could presume that the release of nitrogen-containing products (e.g. proteins) from fungal cells into the liquid medium was responsible for this behavior.

The final observation of the study concerned biomass production in the tested variants (Fig. 7). The morphological change from pelleted to clumped and loose morphology recorded at 20 g l^−1^ of YE resulted in the highest biomass production noted in the experiment, equal to 7 g l^−1^. Applying 200 g l^−1^ of LAC led to a visibly lower level of biomass (4.5 g l^−1^). Finally, using less than 20 g l^−1^ of LAC or less than 2 g l^−1^ of YE led to aggravated biomass growth compared to the media with a higher concentration of substrates.

The C/N ratio was previously claimed to be a significant factor influencing the growth and metabolism of *A. terreus* (Casas Lopez et al. 2003). The present study greatly expanded the range of tested C/N values and proved that having an equivalent C/N value, as well as the same type of C and N sources in the medium is still insufficient to expect similar metabolic and morphological outcomes when comparing cultivation processes. It is now clear that not only the C/N ratio itself but also the way it is achieved (variations in carbon and nitrogen sources concentrations) are extremely important in this context. For example, in the present study the C/N value equal to 374 corresponded to two tested media, yet the results recorded for these experiments were completely different despite the equivalent C/N. Finally, it was demonstrated here that at the C/N values as low as 4 and as high as 374 *A. terreus* continued to exhibit growth and the production of secondary metabolites.

The present work is not the first study reporting the morphological peculiarities of *A. terreus*. In the context of pathogenicity, this species was shown to be capable of forming two distinct types of conidia during the infection, namely the phialidic and accessory conidia, which induce different immunological responses. Moreover, the distribution of accessory conidia on the colonizing hyphae was demonstrated to follow two distinct patterns (i.e. thorn-like or opposing) (see Bengyella et al. (2017) and references therein). The remarkable morphological adaptability displayed by this species is believed to be important in terms of invading the host (Vyas 2011). Analogously, the morphological transitions of *A. terreus* demonstrated in the present work can be viewed as the display of fungal adaptational capabilities under the conditions of submerged cultivation.

## Conclusions

Applying extremely high concentration of yeast extract leads to marked morphological changes of *A. terreus*, namely the growth in the form of clumps and loose mycelium instead of pellets typically observed under submerged conditions. It also results in the complete elimination of (+)-geodin and asterric acid production, as well as relatively low titers of lovastatin. By contrast, using the medium containing an extremely high level of lactose completely blocks the formation of butyrolactone I, stimulates lovastatin production and, at the same time, enhances (+)-geodin and asterric acid biosynthesis. The extremely low supply of yeast extract or lactose does not make it impossible for the pellets to develop, albeit they are visibly smaller than in the richer media. Importantly, at the C/N values as low as 4 and as high as 374 the growth and secondary metabolite production can still be observed in *A. terreus* cultures. The study demonstrated that the media of equivalent C/N ratios may lead to markedly different metabolic and morphological outcomes depending on the concentration of carbon and nitrogen sources that contribute to the C/N value of the given medium.

## Electronic supplementary material

Below is the link to the electronic supplementary material.

Supplementary file1 (PDF 489 kb)
