# Supplementary material for: Exploring the extremes: applying high concentration of yeast extract leads to drastic morphological changes and elimination of (+)-geodin and asterric acid production in Aspergillus terreus submerged cultures
Source: Biotechnol Lett. 2020 Oct 7;43(1):61–71. doi: 10.1007/s10529-020-03018-5 (PMC7796888; doi:10.1007/s10529-020-03018-5)
Supplement: Supplementary file 1 — Supplementary file1 (PDF 489 kb) [file 10529_2020_3018_MOESM1_ESM.pdf]

Supplementary material

Biotechnology Letters

“Exploring the extremes: Applying high concentration of yeast extract leads to drastic morphological changes and elimination of (+)-geodin and asterric acid production in *Aspergillus terreus* submerged cultures”

Tomasz Boruta*, Adrianna Górnicka, Iwona Grzybowska, Ida Stefaniak, Marcin Bizukojc

Lodz University of Technology, Faculty of Process and Environmental Engineering, Department of Bioprocess Engineering, ul. Wolczanska 213, 90-924 Lodz, Poland

* Corresponding author. Phone: +48 42 631 39 77; fax: +48 42 636 56 63.

E-mail address: tomasz.boruta@p.lodz.pl

Supplementary Table 1: Results of two-sample t-test (significance level α = .05) performed to evaluate, whether the mean results of projected area (reported in fig. 3a) obtained for the standard cultivation medium (containing 20 g l^{-1} of lactose and 2 g l^{-1} of yeast extract) differed significantly from the mean values recorded for other tested media at a given time of the process (LAC, lactose; YR, yeast extract; NS, not significant)

Cultivation time [h]	*P* values recorded for the tested growth media					
	LAC 0.2 g l^{-1} YE 2 g l^{-1}	LAC 2 g l^{-1} YE 2 g l^{-1}	LAC 200 g l^{-1} YE 2 g l^{-1}	LAC 20 g l^{-1} YE 0.2 g l^{-1}	LAC 20 g l^{-1} YE 20 g l^{-1}	LAC 20 g l^{-1} YE 200 g l^{-1}
24	NS*	NS*	<.001	<.001	<.001	<.001
48	<.001	<.001	<.001	<.001	<.001	<.001
72	<.001	<.001	<.001	<.001	<.001	<.001
96	<.001	<.001	<.001	<.001	<.001	<.001
120	<.001	NS*	<.001	<.001	<.001	<.001
144	<.001	NS*	<.001	<.001	<.001	<.001
168	< .001	NS*	<.001	<.001	<.001	<.001

*P >.05

Supplementary Table 2: Results of two-sample t-test (significance level α = .05) performed to evaluate, whether the mean results of elongation (reported in fig. 3b) obtained for the standard cultivation medium (containing 20 g l^{-1} of lactose and 2 g l^{-1} of yeast extract) differed significantly from the mean values recorded for other tested media at a given time of the process (LAC, lactose; YR, yeast extract; NS, not significant)

Cultivation time [h]	*P* values recorded for the tested growth media					
	LAC 0.2 g l^{-1} YE 2 g l^{-1}	LAC 2 g l^{-1} YE 2 g l^{-1}	LAC 200 g l^{-1} YE 2 g l^{-1}	LAC 20 g l^{-1} YE 0.2 g l^{-1}	LAC 20 g l^{-1} YE 20 g l^{-1}	LAC 20 g l^{-1} YE 200 g l^{-1}
24	NS*	NS*	NS*	<.001	NS*	<.001
48	NS*	NS*	NS*	<.001	<.001	<.001
72	NS*	NS*	NS*	<.001	NS*	<.001
96	.003	<.001	NS*	<.001	NS*	<.001
120	<.001	<.001	NS*	<.001	.012	<.001
144	<.001	.007	NS*	<.001	.029	<.001
168	.003	NS*	<.001	<.001	<.001	<.001

*P >.05

Supplementary Table 3: Results of two-sample t-test (significance level α = .05) performed to evaluate, whether the mean results of convexity (reported in fig. 3c) obtained for the standard cultivation medium (containing 20 g l^{-1} of lactose and 2 g l^{-1} of yeast extract) differed significantly from the mean values recorded for other tested media at a given time of the process (LAC, lactose; YR, yeast extract; NS, not significant)

Cultivation time [h]	*P* values recorded for the tested growth media					
	LAC 0.2 g l^{-1} YE 2 g l^{-1}	LAC 2 g l^{-1} YE 2 g l^{-1}	LAC 200 g l^{-1} YE 2 g l^{-1}	LAC 20 g l^{-1} YE 0.2 g l^{-1}	LAC 20 g l^{-1} YE 20 g l^{-1}	LAC 20 g l^{-1} YE 200 g l^{-1}
24	NS*	.002	<.001	<.001	<.001	<.001
48	<.001	.023	<.001	<.001	<.001	<.001
72	NS*	<.001	NS*	<.001	<.001	<.001
96	<.001	NS*	<.001	<.001	<.001	<.001
120	<.001	<.001	<.001	<.001	<.001	<.001
144	<.001	<.001	<.001	<.001	<.001	<.001
168	<.001	<.001	.009	<.001	NS*	<.001

*P >.05

Supplementary Table 4: Results of two-sample t-test (significance level α = .05) performed to evaluate, whether the mean results of circularity (reported in fig. 3d) obtained for the standard cultivation medium (containing 20 g l^{-1} of lactose and 2 g l^{-1} of yeast extract) differed significantly from the mean values recorded for other tested media at a given time of the process (LAC, lactose; YR, yeast extract; NS, not significant)

Cultivation time [h]	*P* values recorded for the tested growth media					
	LAC 0.2 g l^{-1} YE 2 g l^{-1}	LAC 2 g l^{-1} YE 2 g l^{-1}	LAC 200 g l^{-1} YE 2 g l^{-1}	LAC 20 g l^{-1} YE 0.2 g l^{-1}	LAC 20 g l^{-1} YE 20 g l^{-1}	LAC 20 g l^{-1} YE 200 g l^{-1}
24	NS*	NS*	<.001	<.001	<.001	<.001
48	<.001	NS*	<.001	<.001	<.001	NS*
72	NS*	NS*	NS*	<.001	<.001	<.001
96	<.001	NS*	<.001	<.001	<.001	NS*
120	NS*	.011	NS*	.002	<.001	NS*
144	NS*	NS*	.006	NS*	.008	NS*
168	<.001	<.001	<.001	<.001	<.001	<.001

*P >.05

Supplementary Table 5: Results of two-sample t-test (significance level α = .05) performed to evaluate, whether the mean results of lovastatin concentration (reported in fig. 5a) obtained for the standard cultivation medium (containing 20 g l^{-1} of lactose and 2 g l^{-1} of yeast extract) differed significantly from the mean values recorded for other tested media at a given time of the process (LAC, lactose; YR, yeast extract; NS, not significant)

Cultivation time [h]	*P* values recorded for the tested growth media					
	LAC 0.2 g l^{-1} YE 2 g l^{-1}	LAC 2 g l^{-1} YE 2 g l^{-1}	LAC 200 g l^{-1} YE 2 g l^{-1}	LAC 20 g l^{-1} YE 0.2 g l^{-1}	LAC 20 g l^{-1} YE 20 g l^{-1}	LAC 20 g l^{-1} YE 200 g l^{-1}
0	NS*	NS*	NS*	NS*	NS*	NS*
24	<.001	.048	.002	<.001	<.001	<.001
48	<.001	<.001	<.001	<.001	<.001	<.001
72	<.001	<.001	.002	<.001	<.001	<.001
96	<.001	<.001	.009	<.001	<.001	<.001
120	<.001	<.001	<.001	<.001	<.001	<.001
144	<.001	<.001	.002	<.001	<.001	<.001
168	<.001	<.001	.002	<.001	<.001	<.001

**P* >.05

Supplementary Table 6: Results of two-sample t-test (significance level α = .05) performed to evaluate, whether the mean results of (+)-geodin concentration (reported in fig. 5b) obtained for the standard cultivation medium (containing 20 g l^{-1} of lactose and 2 g l^{-1} of yeast extract) differed significantly from the mean values recorded for other tested media at a given time of the process (LAC, lactose; YR, yeast extract; NS, not significant)

Cultivation time [h]	*P* values recorded for the tested growth media					
	LAC 0.2 g l^{-1} YE 2 g l^{-1}	LAC 2 g l^{-1} YE 2 g l^{-1}	LAC 200 g l^{-1} YE 2 g l^{-1}	LAC 20 g l^{-1} YE 0.2 g l^{-1}	LAC 20 g l^{-1} YE 20 g l^{-1}	LAC 20 g l^{-1} YE 200 g l^{-1}
0	NS*	NS*	NS*	NS*	NS*	NS*
24	NS*	NS*	NS*	NS*	NS*	NS*
48	NS*	NS*	NS*	NS*	NS*	NS*
72	NS*	NS*	NS*	NS*	NS*	NS*
96	NS*	NS*	NS*	NS*	NS*	NS*
120	NS*	NS*	NS*	NS*	NS*	NS*
144	<.001	<.001	.003	<.001	<.001	<.001
168	.007	.007	.008	.012	.007	.007

**P* >.05

Supplementary Table 7: Results of two-sample t-test (significance level α = .05) performed to evaluate, whether the mean results of butyrolactone I concentration (reported in fig. 5c) obtained for the standard cultivation medium (containing 20 g l^{-1} of lactose and 2 g l^{-1} of yeast extract) differed significantly from the mean values recorded for other tested media at a given time of the process (LAC, lactose; YR, yeast extract; NS, not significant)

Cultivation time [h]	*P* values recorded for the tested growth media					
	LAC 0.2 g l^{-1} YE 2 g l^{-1}	LAC 2 g l^{-1} YE 2 g l^{-1}	LAC 200 g l^{-1} YE 2 g l^{-1}	LAC 20 g l^{-1} YE 0.2 g l^{-1}	LAC 20 g l^{-1} YE 20 g l^{-1}	LAC 20 g l^{-1} YE 200 g l^{-1}
0	NS*	NS*	NS*	NS*	NS*	NS*
24	NS*	NS*	NS*	NS*	NS*	NS*
48	.031	.032	<.001	<.001	.016	<.001
72	.030	<.001	<.001	<.001	NS*	<.001
96	NS*	.003	.007	.007	ns	.007
120	.024	.002	<.001	<.001	ns	.002
144	.009	<.001	.003	.003	ns	NS*
168	.024	.005	.010	.010	ns	.018

*P >.05

Supplementary Table 8: Results of two-sample t-test (significance level α = .05) performed to evaluate, whether the mean results of asterric acid concentration (reported in fig. 5d) obtained for the standard cultivation medium (containing 20 g l^{-1} of lactose and 2 g l^{-1} of yeast extract) differed significantly from the mean values recorded for other tested media at a given time of the process (LAC, lactose; YR, yeast extract; NS, not significant)

Cultivation time [h]	*P* values recorded for the tested growth media					
	LAC 0.2 g l^{-1} YE 2 g l^{-1}	LAC 2 g l^{-1} YE 2 g l^{-1}	LAC 200 g l^{-1} YE 2 g l^{-1}	LAC 20 g l^{-1} YE 0.2 g l^{-1}	LAC 20 g l^{-1} YE 20 g l^{-1}	LAC 20 g l^{-1} YE 200 g l^{-1}
0	NS*	NS*	NS*	NS*	NS*	NS*
24	NS*	NS*	NS*	NS*	NS*	NS*
48	NS*	NS*	NS*	NS*	NS*	NS*
72	NS*	NS*	NS*	NS*	NS*	NS*
96	NS*	NS*	NS*	NS*	NS*	NS*
120	.004	.004	NS*	.008	.004	.004
144	.002	.002	.048	NS*	.002	.002
168	<.001	<.001	.008	NS*	<.001	<.001

*P >.05

Supplementary Table 9: Results of two-sample t-test (significance level α = .05) performed to evaluate, whether the mean results of lactose concentration (reported in fig. 6a) obtained for the standard cultivation medium (containing 20 g l^{-1} of lactose and 2 g l^{-1} of yeast extract) differed significantly from the mean values recorded for other tested media at a given time of the process (LAC, lactose; YR, yeast extract; NS, not significant)

Cultivation time [h]	*P* values recorded for the tested growth media					
	LAC 0.2 g l^{-1} YE 2 g l^{-1}	LAC 2 g l^{-1} YE 2 g l^{-1}	LAC 200 g l^{-1} YE 2 g l^{-1}	LAC 20 g l^{-1} YE 0.2 g l^{-1}	LAC 20 g l^{-1} YE 20 g l^{-1}	LAC 20 g l^{-1} YE 200 g l^{-1}
0	<.001	<.001	<.001	NS*	NS*	NS*
24	<.001	<.001	<.001	NS*	.026	.008
48	<.001	<.001	<.001	.022	.049	.001
72	<.001	<.001	<.001	.012	.022	.006
96	<.001	<.001	<.001	<.001	NS*	NS*
120	<.001	<.001	<.001	<.001	.038	NS*
144	<.001	<.001	<.001	<.001	.023	NS*
168	.003	.003	<.001	<.001	.004	.032

**P* >.05

Supplementary Table 10: Results of two-sample t-test (significance level α = .05) performed to evaluate, whether the mean results of total nitrogen concentration (reported in fig. 6b) obtained for the standard cultivation medium (containing 20 g l^{-1} of lactose and 2 g l^{-1} of yeast extract) differed significantly from the mean values recorded for other tested media at a given time of the process (LAC, lactose; YR, yeast extract; NS, not significant)

Cultivation time [h]	*P* values recorded for the tested growth media					
	LAC 0.2 g l^{-1} YE 2 g l^{-1}	LAC 2 g l^{-1} YE 2 g l^{-1}	LAC 200 g l^{-1} YE 2 g l^{-1}	LAC 20 g l^{-1} YE 0.2 g l^{-1}	LAC 20 g l^{-1} YE 20 g l^{-1}	LAC 20 g l^{-1} YE 200 g l^{-1}
0	NS*	NS*	NS*	<.001	<.001	<.001
24	NS*	NS*	NS*	<.001	<.001	<.001
48	.006	.031	NS*	.017	<.001	<.001
72	<.001	.002	NS*	.025	<.001	<.001
96	<.001	<.001	.029	.014	<.001	<.001
120	<.001	<.001	.011	<.001	<.001	<.001
144	<.001	.002	NS*	.012	<.001	<.001
168	<.001	<.001	NS*	<.001	<.001	<.001

**P* >.05

Supplementary Table 11: Results of two-sample t-test (significance level α = .05) performed to evaluate, whether the mean results of biomass production (reported in fig. 7) obtained for the standard cultivation medium (containing 20 g l^{-1} of lactose and 2 g l^{-1} of yeast extract) differed significantly from the mean values recorded for other tested media at 168 h of the process (LAC, lactose; YR, yeast extract)

Cultivation time [h]	*P* values recorded for the tested growth media					
	LAC 0.2 g l^{-1} YE 2 g l^{-1}	LAC 2 g l^{-1} YE 2 g l^{-1}	LAC 200 g l^{-1} YE 2 g l^{-1}	LAC 20 g l^{-1} YE 0.2 g l^{-1}	LAC 20 g l^{-1} YE 20 g l^{-1}	LAC 20 g l^{-1} YE 200 g l^{-1}
168	<.001	.003	.017	<.001	.009	.004
